# Alcohol harms: the need for prevention and treatment

**DOI:** 10.1093/eurpub/ckag136

**Published:** 2026-07-21

**Authors:** Jørgen G Bramness

**Affiliations:** Institute of Clinical Medicine, UiT – The Arctic University of Norway, Tromsø, Norway; Section for Clinical Addiction Research, Oslo University Hospital, Oslo, Norway; Research Centre for Substance Use Disorders and Mental Illness, Innlandet Hospital Trust, Brumunddal, Norway

Alcohol use in Europe is high and among the highest in the world [1]. Consequently, and in line with a total consumption model for alcohol, we have a high burden of disease following alcohol use on our continent. WHO figures indicate that around 10% of the adult population in Europe have an Alcohol Use Disorder (AUD), with a little more than half of these qualify to alcohol dependence [2]. In Europe alone, more than 800 000 people die each year due to alcohol-related causes, including both acute and chronic conditions that are directly or indirectly linked to alcohol consumption [1]. These alcohol-associated morbidities place a substantial burden on health services, which are already struggling to meet the increasing demand for care.

Beyond the medical consequences, which include cardiovascular diseases and injuries, cancers, and liver disease, alcohol use also generates significant mental, sociological, and economic harms. There is a solid and causal link between alcohol use and mental disorders [2]. As a psychiatric epidemiologist, I am concerned that a considerable proportion of anxiety, insomnia, and depression observed in the general population is, at least in part, attributable to alcohol use. On the societal level, alcohol is strongly implicated in domestic violence and adverse childhood experiences, with lifelong consequences. The economic burden is equally substantial: short-term sickness absences, reduced productivity, and compromised workplace safety due to hangovers represent major costs to employers and to society at large.

The alcohol industry is one of the world’s largest and most profitable industries. The global alcoholic drinks market size was estimated at USD almost 1.9 trillion in 2025 [3]. The industry plays a powerful role in shaping public perceptions of alcohol use. Arguments such as “people can look after themselves” or “we do not need a nanny state” are often strongly aligned with industry interests. The industry consistently opposes population-level alcohol control measures, including restrictions on sales, shorter opening hours, higher taxation, and advertising bans. Its promotional efforts aim to embed alcohol deeply in cultural notions of the “good life,” emphasizing drinking as integral to celebrations, leisure, and socializing. Politicians seeking easy popularity often adopt liberal alcohol policies, and this tendency can be seen in debates on deregulating the alcohol market, such as discussions about dismantling the monopoly in Finland, expanding opening hours in various countries, or the highly publicized alcohol policy debates that accompanied the recent FIFA World Cup.

In view of these pressures, several organizations and initiatives are actively advocating stronger alcohol policies. The European Health Alliance on Alcohol (EHAA) (https://www.who.int/europe/news/item/08-05-2025-european-health-alliance-on-alcohol-launched-to-reduce-the-unsustainable-toll-of-alcohol-harms-in-europe) in collaboration with EU and WHO and the European Alcohol Policy Alliance (https://www.eurocare.org/) collaborate with numerous professional societies to highlight the growing influence of the alcohol industry and the rising burden of alcohol-related harm in Europe. Such initiatives are of high value to try to counteract the industry’s influence on policies and perceptions.

While some countries issue recommended limits for “low-risk” drinking, these limits vary widely—for example, from 40 grams per day in Italy to 12 grams per day in the Netherlands. Recently, Canada introduced one of the lowest recommended thresholds ever, at only 2.5 grams per day, reflecting an emerging scientific consensus that no level of alcohol consumption is truly safe. In line with the WHO, Norway has avoided setting a specific limit to reinforce the message that, from a health perspective, there is no safe level of alcohol consumption. If people wish to avoid adverse health consequences entirely, abstaining from alcohol is the only option. This is not a call for universal abstinence—many aspects of life involve balancing risk—but it is important to acknowledge that alcohol provides no health benefits. Claims about cardiovascular benefits of low-dose drinking originate from studies with methodological issues and should not be interpreted as evidence of protective effects [4].

Despite the high prevalence of AUD treatment rates remain low. Only between 5% and 10% of the population in many countries that meet criteria for AUD are in treatment [2, 5]. This huge treatment gap may be due to appropriate services being unavailable or insufficiently resourced or people not seeking help because they do not think it helps or that they think their treatment goals do not align with what is offered [6].

Recently, the US medicines authorities, the Food and Drug Administration (FDA) approved reduction in drinking as a valid outcome for AUD trials [7]. This treatment goal has long been acknowledged in Europe, but this decision still marks a change in how we view AUD and its trajectory. At least for some people it is possible to return to controlled drinking after having suffered from AUD. This is just one example of how treatment in the AUD field can expand its coverage and move forward.

The field of alcohol prevention and treatment can learn from advances in cardiovascular health. Over the past generation, Europe has witnessed a dramatic reduction in cardiovascular disease—especially coronary heart disease—driven primarily by two developments: a major decline in smoking and substantial improvements in the treatment of cardiac conditions [8, 9]. Together, success in prevention and treatment have contributed significantly to increased life expectancy, particularly among men.

This experience underscores the need for a dual strategy even in the alcohol field. First, we must implement effective population-level regulations that reduce the availability and harmful use of alcohol. Second, we must expand and improve treatment services to close the large treatment gap in AUD. Only through combining strong public health policies with accessible, evidence-based clinical interventions can we hope to reduce the considerable burden of alcohol-related harm in Europe.

Conflict of interest: None declared.
